# Comparative Study of *Sargassum fusiforme* Polysaccharides in Regulating Cecal and Fecal Microbiota of High-Fat Diet-Fed Mice

**DOI:** 10.3390/md19070364

**Published:** 2021-06-24

**Authors:** Bin Wei, Qiao-Li Xu, Bo Zhang, Tao-Shun Zhou, Song-Ze Ke, Si-Jia Wang, Bin Wu, Xue-Wei Xu, Hong Wang

**Affiliations:** 1 Key Laboratory of Marine Ecosystem and Biogeochemistry, State Oceanic Administration & Second Institute of Oceanography, Ministry of Natural Resources, Hangzhou 310012, China; binwei@zjut.edu.cn; 2 College of Pharmaceutical Science & Collaborative Innovation Center of Yangtze River Delta Region Green Pharmaceuticals, Zhejiang University of Technology, Hangzhou 310014, China; liujiahui0829@163.com (Q.-L.X.); zhangbo_1632021@163.com (B.Z.); zhoutaoshun@yeah.net (T.-S.Z.); kesongzeke@163.com (S.-Z.K.); new8090@hotmail.com (S.-J.W.); 3 Center for Human Nutrition, David Geffen School of Medicine, University of California, Rehabilitation Building 32-21, 1000 Veteran Avenue, Los Angeles, CA 90024, USA; 4 Zhoushan Campus, Ocean College, Zhejiang University, Zhoushan 316021, China; wubin@zju.edu.cn

**Keywords:** *Sargassum fusiforme* polysaccharides, high-fat diet, cecal microbiota, fecal microbiota, 16S rRNA gene sequencing

## Abstract

Seaweed polysaccharides represent a kind of novel gut microbiota regulator. The advantages and disadvantages of using cecal and fecal microbiota to represent gut microbiota have been discussed, but the regulatory effects of seaweed polysaccharides on cecal and fecal microbiota, which would benefit the study of seaweed polysaccharide-based gut microbiota regulator, have not been compared. Here, the effects of two *Sargassum fusiforme* polysaccharides prepared by water extraction (SfW) and acid extraction (SfA) on the cecal and fecal microbiota of high-fat diet (HFD) fed mice were investigated by 16S rRNA gene sequencing. The results indicated that 16 weeks of HFD dramatically impaired the homeostasis of both the cecal and fecal microbiota, including the dominant phyla Bacteroidetes and Actinobacteria, and genera *Coriobacteriaceae*, *S24-7*, and *Ruminococcus*, but did not affect the relative abundance of Firmicutes, *Clostridiales*, *Oscillospira*, and *Ruminococcaceae* in cecal microbiota and the Simpson’s index of fecal microbiota. Co-treatments with SfW and SfA exacerbated body weight gain and partially reversed HFD-induced alterations of *Clostridiales* and *Ruminococcaceae*. Moreover, the administration of SfW and SfA also altered the abundance of genes encoding monosaccharide-transporting ATPase, α-galactosidase, β-fructofuranosidase, and β-glucosidase with the latter showing more significant potency. Our findings revealed the difference of cecal and fecal microbiota in HFD-fed mice and demonstrated that SfW and SfA could more significantly regulate the cecal microbiota and lay important foundations for the study of seaweed polysaccharide-based gut microbiota regulators.

## 1. Introduction

Gut microbiota is a population of microorganisms that colonizes the intestines. This not only protects against pathogens, provides nutrients, and maintains the integrity of the mucosal barrier, but also plays an important role in numerous diseases, such as inflammatory bowel disease, obesity, diabetes mellitus, metabolic syndrome, atherosclerosis, non-alcoholic fatty liver disease, etc. [1,2,3,4]. Owing to sampling difficulties, most studies chose to characterize the gut microbiota composition by sequencing the fecal samples, other than the cecal contents [5,6]. The differences of cecal microbiota and fecal microbiota in mice and human volunteers have been compared by several research groups [7,8,9]. Guo et al. reported that the relative abundance of an unidentified genus from the S24-7 family (*S24-7*) in cecal microbiota was much higher than that in fecal microbiota, and oral administration of a marine carotenoid, fucoxanthin, significantly increased the abundance of *S24-7* in cecal microbiota but decreased the abundance of the genus in fecal microbiota [7]. Stanley et al. found that fecal and cecal microbiotas showed qualitative similarities but quantitative differences [8]. Marteau et al. compared the bacterial compositions within the human cecal and fecal microbiota and found that the abundance of *Bifidobacteria*, *Bacteroides*, *Clostridium coccoides* group, and *Clostridium leptum* subgroup were significantly lower in the cecum [9]. Therefore, comparative studies on the cecal and fecal microbiota in a specific situation are necessary to better understand the characteristics of gut microbiota.

High-fat diet (HFD) feeding has been widely used as a model for studying metabolic syndrome and gut microbiota dysbiosis [10,11]. Seaweed polysaccharides represent a kind of promising natural product that alleviates HFD-induced metabolic syndrome and promotes the healthy growth of gut bacteria [12]. In recent years, polysaccharides prepared from *Sargassum fusiforme*, a well-known edible alga, have attracted extensive research interest due to their potential biomedical application [13,14,15,16,17]. For example, our recent study indicated five polysaccharides prepared from *S*. *fusiforme* could selectively regulate the relative abundance of *Oscillospira* and *Clostridiales* in cecal microbiota of HFD-fed mice [16]. Cheng and colleagues prepared an *S. fusiforme* polysaccharide that could decrease the relative abundances of the diabetes-related fecal microbiota [17]. However, the regulatory effects of *S. fusiforme* polysaccharides on cecal microbiota and fecal microbiota, which would be helpful for the study of seaweed polysaccharide-based gut microbiota regulators, have not been compared.

In this study, two polysaccharides were prepared from *S. fusiforme* by water extraction (SfW) and acid extraction (SfA), and their chemical structures were characterized according to our recent report [16]. Then, the effects of 16 weeks of SfW and SfA administration on the cecal and fecal microbiota of HFD-fed mice were investigated.

## 2. Results

### 2.1. Chemical Structures of Sargassum fusiforme Polysaccharides

The physicochemical properties of the two *S. fusiforme* polysaccharides prepared by water extraction (SfW) and acid extraction (SfA) are shown in Table 1. The results indicated that the chemical structures of SfW and SfA were quite similar. For example, they had comparable contents of total sugar (70.0% vs. 62.4%) and sulfate group (28.5% vs. 31.3%), and their average molecular weights were also very close. Moreover, the monosaccharide compositions of the two polysaccharides were very similar. Both of them were mainly composed of glucose, fucose, and galactose with small amounts of mannose, glucuronic acid, and xylose, but the detailed molar ratio had a slight difference. For example, the content of xylose in SfW was lower than that in SfA (0.14 vs. 0.03), while glucose was more abundant in SfA (1.05 vs. 1.13).

Here, ^1^H NMR spectra of SfW and SfA are shown in Figure S1. The resonance signals of the two polysaccharides at 3.0–5.5 ppm were ascribed to the typical distribution of ^1^H NMR signals of the polysaccharides [18]. The unresolved peaks at 5.3–5.5 ppm were assigned to the anomeric protons of α-l-fucopyranosyl units [19]. The resonance signals of the two polysaccharides at 3.3–4.5 ppm were apportioned to the ring protons H-2 to H-5, but the pattern was different from each other, and the chemical shifts at 1.1 and 1.4 ppm were assigned to methyl groups of fucose units [20]. In addition, due to the complex and heterogeneous structure of sulfated polysaccharides, the broadening and overlapping of ^1^H NMR peaks makes it difficult to completely describe their structural characteristics.

### 2.2. Effects of SfW and SfA on HFD-Induced Metabolic Disorders

**Figure 1:**
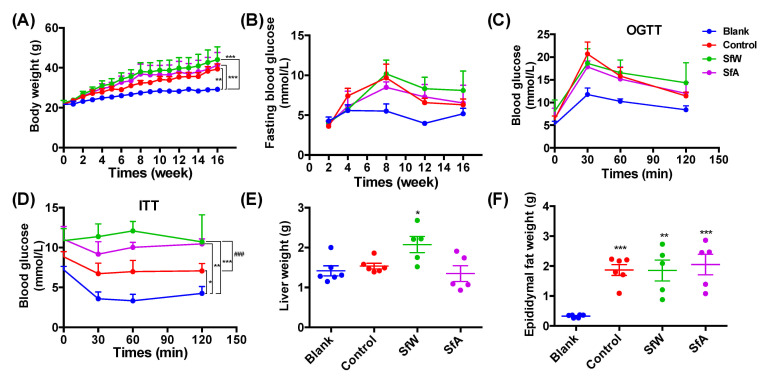
Effects of SfW and SfA on (**A**) body weight, (**B**) fasting blood glucose, (**C**) OGTT, (**D**) ITT, (**E**) liver weight, and (**F**) epididymal fat weight in HFD-fed mice. Values are mean ± SD (*n* = 5–6). * *p* < 0.05, ** *p* < 0.01, *** *p* < 0.001 vs. Blank. ### *p* < 0.001 vs. Control. Data sets in (**A**–**D**) were analyzed using unpaired two-tailed Student’s *t*-test. Data sets in (**E**–**F**) were analyzed using one-way ANOVA followed by a Turkey’s test.

The effects of SfW and SfA on HFD-induced metabolic disorders were evaluated after 16 weeks of co-treatment with HFD and polysaccharides. As shown in Figure 1A, 16-week HFD feeding significantly increased the body weight of the mice compared to that of the blank group, and co-treatment with SfW and SfA exacerbated body weight gain. The fasting blood glucose level significantly increased after 4 weeks of HFD-feeding, and only treatment with SfW at the fourth week reversed the increase (*p* < 0.05) (Figure 1B). In OGTT, the blood glucose reached the maximal level at 30 min after the dextrose gavage, and the blood glucose level of the control group was significantly higher than that of the blank group (*p* < 0.001). The polysaccharides administration could not attenuate the HFD-induced glucose intolerance (Figure 1C). Treatments with SfW and SfA did not protect HFD-induced insulin resistance and epididymal fat weight gain (Figure 1D–F).

**Figure 2:**
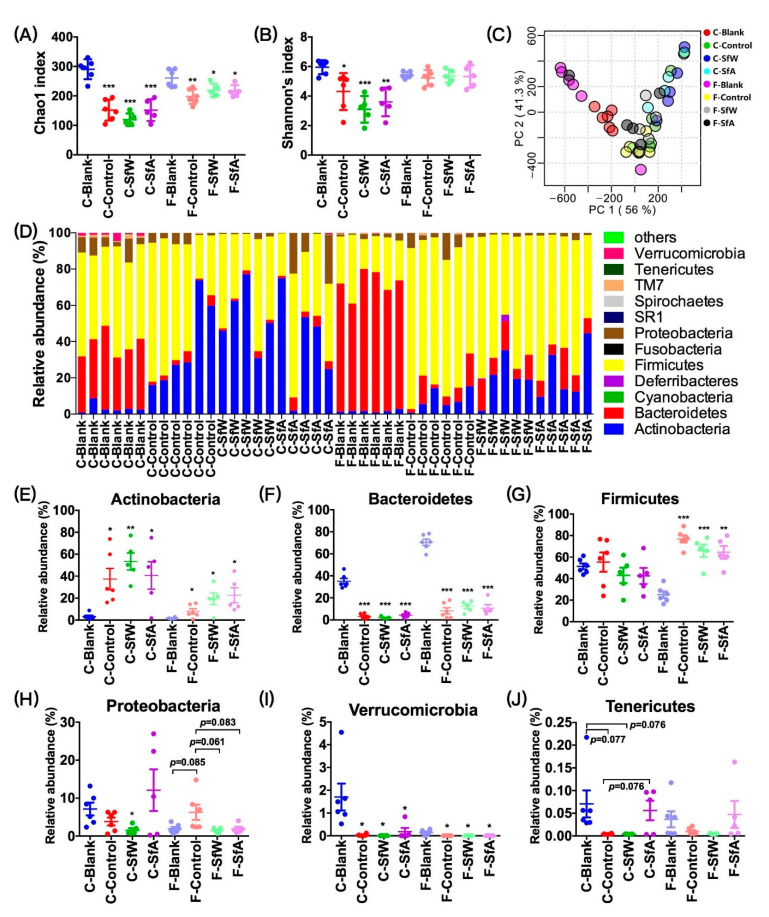
Effects of SfW and SfA on the (**A**) Chao1 diversity and (**B**) Shannon’s diversity indices of cecal and fecal microbiota in HFD-fed mice. (**C**) PCA and (**D**) bar plots of cecal and fecal microbiota at the phylum level. The relative abundance of (**E**) Actinobacteria, (**F**) Bacteroidetes, (**G**) Firmicutes, (**H**) Proteobacteria, (**I**) Verrucomicrobia, and (**J**) Tenericutes in cecal and fecal microbiota. Values are mean ± SEM. * *p* < 0.05, ** *p* < 0.01, *** *p* < 0.001 vs. the corresponding blank group. Data sets in (**A**), (**B**), (**E**–**J**) were analyzed using one-way ANOVA followed by a Turkey’s test. C-, cecal microbiota; F-, fecal microbiota.

The effects of 16 weeks of HFD and polysaccharide administration on gut microbiota in fecal samples and cecal contents of mice were analyzed by 16S rRNA high-throughput sequencing. As shown in Figure 2A–B, HFD significantly decreased both the Chao1 and Simpson’s indices of cecal microbiota but showed negligible effect on the Simpson’s index of fecal microbiota. Oral administration of SfW and SfA did not significantly improve the decrease in α-diversity of fecal and cecal microbiota. The unsupervised principal components analysis (PCA) plot at the phylum level showed that PC1 and PC2 were able to explain 56% and 41.3% of the variation, respectively, and exhibited significant distinction between the cecal and fecal microbiota of the blank group (Figure 2C). Polysaccharide administration showed no significant regulatory effect on the dysbiosis of cecal and fecal microbiota at the phylum level (Figure 2C). In detail, HFD significantly increased the relative abundance of Actinobacteria and decreased the abundance of Bacteroidetes and Verrucomicrobia in both the cecal and fecal microbiota but only enriched Firmicutes and Proteobacteria in fecal microbiota. SfW only presented a regulatory effect on Proteobacteria in fecal microbiota (Figure 2H,J).

**Figure 3:**
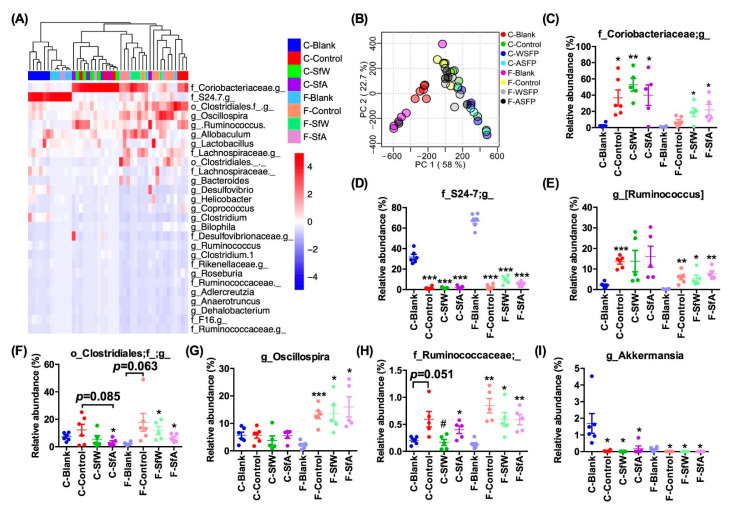
Effects of SfW and SfA on the cecal and fecal microbiota in HFD-fed mice. (**A**) Heatmap and (**B**) PCA plots of cecal and fecal microbiota at the genus level. The relative abundance of (**C**) *Coriobacteriaceae*, (**D**) *S24-7*, (**E**) *Ruminococcus*, (**F**) *Clostridiales*, (**G**) *Oscillospira*, (**H**) *Ruminococcaceae*, and (**I**) *Akkermansia* in cecal and fecal microbiota. Values are mean ± SEM. * *p* < 0.05, ** *p* < 0.01, *** *p* < 0.001 vs. the corresponding blank group. # *p* < 0.05 vs. the corresponding control group. Data sets in (**C**–**I**) were analyzed using one-way ANOVA followed by a Turkey’s test. C-, cecal microbiota; F-, fecal microbiota.

The effects of SfW and SfA on the cecal and fecal microbiota in HFD-fed mice were also investigated at the genus level. As shown in Figure 3A, bacteria in these cecal and fecal samples mainly consisted of 27 genera, including *Coriobacteriaceae* and *S24-7*. However, the detailed composition of them between the cecal and fecal microbiota and between the blank and control groups were different. The far distance between the F-Blank group and F-SfW or F-SfA group in the clustering scheme suggested that HFD-induced dysbiosis of cecal microbiota could be more significantly regulated by SfW and SfA. The PCA plot at the genus level further confirmed the compositional difference between the cecal and fecal samples (Figure 3B). HFD mainly altered the relative abundance of *Coriobacteriaceae*, *S24-7*, *Ruminococcus*, *Clostridiales*, *Oscillospira*, *Ruminococcaceae*, and *Akkermansia* in both the cecal and fecal microbiota but only enriched *Oscillospira* in fecal microbiota (Figure 3C–I). Notably, oral administration of SfW and SfA could partially alleviate the increase of *Clostridiales* and *Ruminococcaceae* in fecal microbiota (Figure 3F,H).

### 2.3. Effects of SfW and SfA on the Abundance of Genes Encoding Carbohydrate-Metabolizing Enzymes in Cecal and Fecal Microbiota

**Figure 4:**
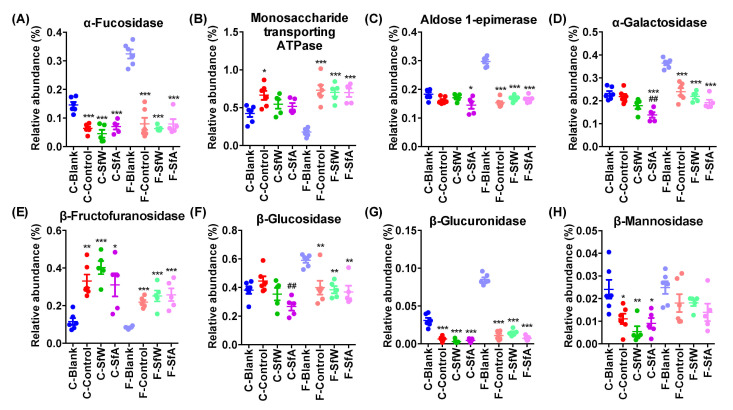
Effects of SfW and SfA on functional gene of cecal and fecal microbiota in HFD-fed mice. (**A**) α-Fucosidase, (**B**) monosaccharide-transporting ATPase, (**C**) aldose 1-epimerase, (**D**) α-galactosidase, (**E**) β-fructofuranosidase, (**F**) β-glucosidase, (**G**) β-glucuronidase, and (**H**) β-mannosidase. Values are mean ± SEM. * *p* < 0.05, ** *p* < 0.01, *** *p* < 0.001 vs. the corresponding blank group. ## *p* < 0.01 vs. the corresponding control group. Data sets were analyzed using one-way ANOVA followed by a Turkey’s test. C-, cecal microbiota; F-, fecal microbiota.

The effects of *S. fusiforme* polysaccharides on the abundance of genes encoding carbohydrate-metabolizing enzymes in the cecal and fecal microbiota of HFD-fed mice were investigated using PICRUSt2 based on the 16S rRNA gene sequencing data. The results demonstrated that the HFD significantly decreased the abundance of genes encoding α-fucosidase (Figure 4A) and β-glucuronidase (Figure 4G), and increased that of monosaccharide-transporting ATPase (Figure 4B) and β-fructofur-anosidase (Figure 4E) in both the cecal and fecal microbiota, but the alterations of genes encoding α-galactosidase (Figure 4D) and β-glucosidase (Figure 4F) were only observed in fecal microbiota, and that of β-mannosidase (Figure 4H) was only presented in cecal microbiota. The administration of SfW and SfA mainly regulated the abundance of genes encoding monosaccharide-transporting ATPase, α-galactosidase, β-fructofuranosidase, and β-glucosidase with the latter showed more significant potency. For example, SfA alleviated the increase of genes encoding monosaccharide-transporting ATPase and β-glucosidase (Figure 4B–F).

## 3. Discussion

Seaweed polysaccharides have various biological activities [11,13,21,22,23,24,25] and also show potential to be developed as prebiotics that promote the healthy growth of gut bacteria. Previous studies have compared the similarities and differences between the cecal and fecal microbiota of animals and human volunteers [6,7,8]. However, the regulatory effects of polysaccharides extracted from *S. fusiforme* (a common and widely eaten seaweed) on the cecal and fecal microbiota of HFD-fed mice, which would benefit the study of seaweed polysaccharide-based gut microbiota regulators, have not been compared. Here, the impacts of 16 weeks of water and acid extracted *S. fusiforme* polysaccharides (SfW and SfA) on the cecal and fecal microbiota of HFD-fed mice were investigated. We found that the HFD significantly altered the dominant phyla Bacteroidetes and Actinobacteria, and the dominant genera *Coriobacteriaceae*, *S24-7*, and *Ruminococcus*, but did not affect the abundance of Firmicutes, *Clostridiales*, *Oscillospira*, and *Ruminococcaceae* in cecal microbiota and the Simpson’s index of fecal microbiota. Co-treatments with SfW and SfA partially reversed the dysbiosis of Firmicutes, *S24-7*, *Ruminococcus*, *Clostridiales*, and *Ruminococcaceae*. The administration of SfW and SfA also altered the abundance of some genes encoding carbohydrate-metabolizing enzymes between cecal and fecal microbiota in 16-week HFD-fed mice and demonstrated that cecal microbiota was more significantly regulated by *S. fusiforme* polysaccharides.

Our recent study reported that five *S*. *fusiforme* polysaccharides prepared through hot-water and acid extraction showed a wide range of molecular weight (10–698.3 kDa), and the HCl-extracted polysaccharide (Sf-A) mainly consisted of glucose, fucose, and galactose [16]. Cheng et al. prepared a 205.8 kDa *S*. *fusiforme* polysaccharide (SFF) by acid extraction and the polysaccharide was mainly composed of fucose and galactose [17]. The chemical structures of SfW and SfA were more similar to Sf-A than SFF, which may be because they are sourced from the same supplier.

HFD has been demonstrated to adversely affect gut microbiota composition through increasing the abundance of Firmicutes and Proteobacteria and decreasing Bacteroidetes [26,27,28,29]. The present study demonstrated that 16 weeks of HFD significantly increased the relative abundance of Actinobacteria and decreased the abundance of Bacteroidetes and Verrucomicrobia in both the cecal and fecal microbiota but only enriched Firmicutes and Proteobacteria in fecal microbiota. The discrepancy between the previous studies with our findings indicates that HFD consistently decreases the abundance of Bacteroidetes in both the cecal and fecal microbiota, but the impacts on other phyla depend on the sampling position and the specific conditions of the animal models. Our recent study demonstrated that four weeks of HFD feeding significantly increased the relative abundance of *Coriobacteriaceae* and *Oscillospira* in cecal microbiota [11], but in the present study, the abundance of *Oscillospira* in cecal microbiota was not enriched by HFD, which may be explained by the prolonged HFD treatment. These findings further deepen our understanding about the impact of HFD on gut microbiota composition.

It is well known that microbiota composition varies in different parts of the gastrointestinal tract, and there are significant differences in the quantity and quality of microorganisms in the cecum contents and feces [8]. According to a recent report, the cecal and fecal microbiota exhibits different taxonomic structures, functional activities, and metabolic pathways [30]. Guo et al. also reported that the abundance of Firmicutes in the cecum of mice fed a normal diet was much higher than that in the feces, and *S24-7* was much lower in the cecal contents, which was highly consistent with the present study [9]. Here, we comprehensively compared the similarities and differences between the cecal and fecal microbiota in 16-week HFD-fed mice, including the abundance of genes encoding carbohydrate-metabolizing enzymes in cecal and fecal microbiota. HFD showed a more significant influence on the α-diversity of cecal microbiota than that of fecal microbiota, suggesting that cecal microbiota may be more suitable to represent the gut microbiota composition. Some bacteria (Actinobacteria and *Coriobacteriaceae*) were significantly enriched in the cecal contents, while others (Firmicutes, *Oscillospira*, and *Ruminococcaceae*) were enriched in the fecal samples, which may be associated with the different function of the cecum and colon. For example, *Coriobacteriaceae*, a family within the phylum Actinobacteria, are strictly anaerobic bacteria and contribute to the metabolism of bile salts, steroids, and dietary polyphenols [31]. Ariangela et al. reported that the TNBS colitis severity was most closely correlated with the composition of colonic mucus microbiome, but not fecal or cecal microbiome [32], suggesting that the choice of sampling site depends on the experimental design. Interestingly, SfA was mainly composed of glucose, fucose, and galactose, and administration of SfA significantly decreased the abundance of genes encoding α-galactosidase and β-glucosidase, which may be ascribed to the interactions between the polysaccharide and gut microbiota.

Previous studies reported the effects of several *S. fusiforme* polysaccharides on gut microbiota composition [11,17,33]. Chen et al. reported that 12 months of oral administration of an *S. fusiforme* polysaccharide decreased the abundance of the phyla Firmicutes, Proteobacteria, and the genera *Lactobacillus* and *Helicobacter* in small intestinal microbiota [33]. Cheng and colleagues found that 6 weeks of oral administration of an *S. fusiforme* polysaccharide significantly decreased the relative abundances of several diabetes-related microbiota in fecal samples, including *Bilophila*, *Oscillibacter*, and *Mucispirillum* [17]. Our previous study reported that 4 weeks of *S. fusiforme* polysaccharide treatment significantly altered the relative abundance of the phylum Bacteroidetes, and the genera *Oscillospira*, *Mucispirillum*, and *Clostridiales* in cecal microbiota [11]. In the present study, 16 weeks of SfW administration partially reversed HFD-induced alterations of *Clostridiales* and *Ruminococcaceae* in both the cecal and fecal microbiota. The differences in the regulatory effects of *S. fusiforme* polysaccharides on gut micobiota may be explained by the structural difference of polysaccharides, or difference in the animal model, and sampling position. For example, the microbiota composition in the intestinal contents from the ICR mice was investigated by Chen et al., while in the present study, we determined the microbiota composition in fecal and cecal samples from the ICR mice.

## 4. Materials and Methods

### 4.1. Materials

The brown algae *S. fusiforme* was sourced from Qingdao, China, on August, 2018. Standards of monosaccharides and 1-phenyl-3-methyl-5-pyrazolone (PMP) were purchased from Aladdin Chemistry Co., Ltd. (Shanghai, China). Dextran standards were purchased from American Polymer Standards Corporation (Mentor, OH, USA). Other reagents and solvents were of analytical grade.

### 4.2. Preparation of Sargassum fusiforme Polysaccharides

As previously reported, *S. fusiforme* polysaccharides were prepared with slight modifications [11]. Briefly, dry *S. fusiforme* was cut into pieces and pre-treated with 95% ethanol to remove the pigment. Polysaccharides were extracted from the residual materials by hot water for 1 h and 0.1 M HCl at 60 °C for 1 h, respectively. The hot water-extracted polysaccharide was further treated with 0.05 M MgCl_2_ to eliminate the alginate, and ultra-filtered to obtain the SfW. The HCl-extracted crude polysaccharide was dialyzed and precipitated using ethanol to obtain the SfA.

### 4.3. Structural Analysis of Sargassum fusiforme Polysaccharides

The chemical analysis of SfW and SfA was based on previous studies [11]. The total sugar content was measured by the phenol-sulfuric acid method using d-glucose as the standard [34]. The content of sulfate was analyzed with the BaCl_2_-gelation method using Na_2_SO_4_ as the standard [35]. The molecular weight analysis was conducted using High-Performance Size Exclusion Chromatography using a Waters 2487 HPLC system with a refractive index detector 2414 (Waters, Milford, MA, USA). The chromatography conditions refer to previous studies [11]. The molar ratio of monosaccharide and fucose content was determined by the PMP derivatization method with minor modification.

SfW and SfA fraction (100 mg) were dissolved in 0.55 mL of D_2_O (99.9%) followed by centrifugation and lyophilization, and the process was repeated three times. Finally, the resulting polysaccharide was dissolved with 0.55 mL of D_2_O. ^1^H NMR spectra were recorded on AVANCE III NMR 600 MHz spectrometer (Bruker Inc., Billerica, MA, USA) at 25 °C.

### 4.4. Animal Experiments

Twenty-two male C57 mice (Specific-pathogen-free grade, six-week-old) were purchased from SPF (Beijing, China) Biotechnology Co., Ltd. (Beijing, China), and kept at the Animal Center of Zhejiang, University of Technology. All mice were randomly divided into four groups (the blank and control groups: *n* = 6; the SfW and SfA groups: *n* = 5) and fed a normal diet for one week to stabilize all the metabolic conditions. Each group was housed in one standard cage under a condition of 22 ± 1 °C, a humidity of 55 ± 5%, and a 12 h light/dark cycle. From the beginning of the experiment, mice in the control, SfW, and SfA groups were fed with an HFD (TP23300, 60 kcal% fat, Trophic Animal Feed High-Tech Co., Ltd, Nantong, China), and mice in the blank group were still fed a standard lab chow diet. The mice in the SfW and SfA groups had free access to 1 mg/mL of SfW and SfA in drinking water, respectively, for a period of 16 weeks, and the other two groups were treated with sterile water.

During the experiments, the body weight was measured weekly, and fasting blood glucose was measured monthly via tail vein using a glucometer (Johnson and Johnson, New Brunswick, NJ, USA) according to the instruction after fasting for 16 h. A sugar tolerance test (OGTT) and insulin tolerance test (ITT) were measured at week 15 and 16, respectively, and the feces were collected before the mice were dissected. All mice were sacrificed from asphyxiation by carbon dioxide. The liver and epididymal fat were harvested and their weight was measured. The cecum contents were collected and stored at −80 °C. The protocol was approved by the Animal Ethics Committee of the Zhejiang University of Technology, China. All efforts were made to minimize the suffering of the mice.

### 4.5. Gut Microbiota Analysis by 16S rRNA Gene Sequencing

Fecal samples and cecal contents were used for gut microbiota analysis by sequencing the 16S rRNA genes. The DNA extraction, PCR amplification, sequencing, and data analysis were conducted according to our previous study [11].

### 4.6. Statistical Analysis

The significance of the differences between the two groups was assessed using the unpaired two-tailed Student’s *t*-test (Figure 1). Data sets that involved more than two groups were assessed by one-way ANOVA followed by a Turkey’s test (Figure 1, Figure 2, Figure 3 and Figure 4). *p* values and the significance level are indicated in the associated figure legends for each figure. Statistical analysis was performed with SPSS statistics software (Version 19.0).

## 5. Conclusions

In conclusion, we compared the microbiota composition in the cecal and fecal microbiota of 16-week HFD-fed mice and revealed that HFD dramatically altered the abundance of Bacteroidetes and Actinobacteria, and *Coriobacteriaceae*, *S24-7*, and *Ruminococcus* in both the cecal and fecal microbiota but did not affect the relative abundance of Firmicutes, *Clostridiales*, *Oscillospira*, *Ruminococcaceae* in cecal microbiota and the Simpson’s index of fecal microbiota. Co-treatments with SfW and SfA exacerbate body weight gain and partially reverse HFD-induced alterations of *Clostridiales* and *Ruminococcaceae* and alter the abundance of genes encoding monosaccharide-transporting ATPase, α-galactosidase, β-fructofuranosidase, and β-glucosidase. Our findings provide important insights for the study of seaweed polysaccharide-based gut microbiota regulators.

## Figures and Tables

**Table 1:** Physicochemical properties of *Sargassum fusiforme* polysaccharides.

Samples	Total Sugar (%)	Sulfate (%)	Mw (kDa)	Monosaccharide (Molar Ratio) *					
				Man	Glc A	Glc	Gal	Xyl	Fuc
SfW	70.0	28.5	166/5.9	0.07	0.07	1.05	0.41	0.14	1
SfA	62.4	31.3	276/5.8	0.05	0.06	1.13	0.38	0.03	1

* Man, mannose; GlcA, glucuronic acid; Glc, glucose; Gal, galactose; Xyl, xylose; Fuc, fucose.

## Data Availability

The data in this study are available on request from the corresponding author.

## Supplementary Materials

The following are available online at https://www.mdpi.com/article/10.3390/md19070364/s1. Figure S1 ^1^H-NMR spectra of *Sargassum fusiforme* polysaccharides SfW and SfA.
