# Supplementary figures and images for: Comparative Study of Sargassum fusiforme Polysaccharides in Regulating Cecal and Fecal Microbiota of High-Fat Diet-Fed Mice
Source: Mar Drugs. 2021 Jun 24;19(7):364. doi: 10.3390/md19070364 (PMC8303714; doi:10.3390/md19070364)

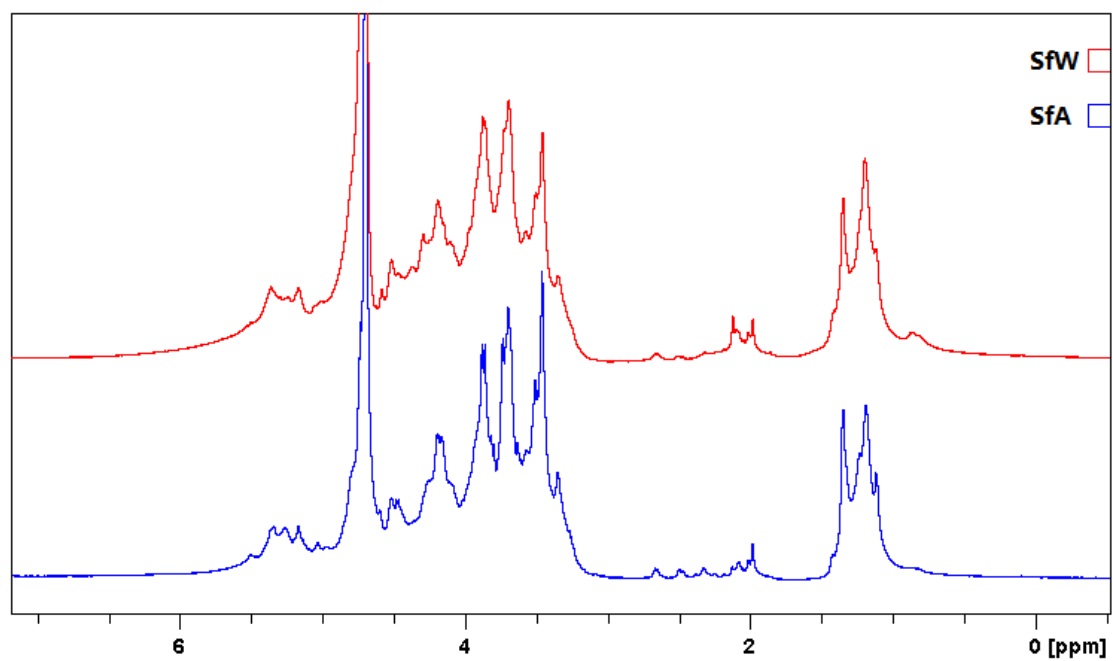

**Fig. S1**  $^1\text{H}$ -NMR spectra of *Sargassum fusiforme* polysaccharides SfW and SfA.

Supplement: Supplementary file 1 [file marinedrugs-19-00364-s001.zip › marinedrugs-1254118-supplementary.pdf]
